# Evaluation of PTSD-Induced Alterations in Bone Biomechanics and the Protective Potential of CE-123 in a Wistar Rat Model

**DOI:** 10.3390/jcm14072427

**Published:** 2025-04-02

**Authors:** Cezary Osiak-Wicha, Katarzyna Kras, Ewa Tomaszewska, Siemowit Muszyński, Paweł Grochecki, Jolanta H. Kotlińska, Tymoteusz Słowik, Michał Świetlicki, Kamil Arciszewski, Gert Lubec, Marcin B. Arciszewski

**Affiliations:** 1 Department of Animal Anatomy and Histology, Faculty of Veterinary Medicine, University of Life Sciences in Lublin, Akademicka 12, 20-950 Lublin, Poland; katarzyna.kras@up.lublin.pl; 2 Department of Animal Physiology, Faculty of Veterinary Medicine, University of Life Sciences in Lublin, Akademicka 12, 20-950 Lublin, Poland; ewa.tomaszewska@up.lublin.pl; 3 Department of Biophysics, Faculty of Environmental Biology, University of Life Sciences in Lublin, Akademicka 13, 20-950 Lublin, Poland; siemowit.muszynski@up.lublin.pl; 4 Department of Pharmacology and Pharmacodynamics, Medical University of Lublin, Chodzki 4a, 20-093 Lublin, Poland; pawel.grochecki@umlub.pl (P.G.); jolanta.kotlinska@umlub.pl (J.H.K.); 5 Experimental Medicine Center, Medical University, Jaczewskiego 8, 20-090 Lublin, Poland; tymoteusz.slowik@umlub.pl; 6 Department of Applied Physics, Faculty of Mechanical Engineering, Lublin University of Technology, Nadbystrzycka 36, 20-618 Lublin, Poland; m.swietlicki@pollub.pl; 7 Clinical Neurology Ward, The University Clinical Hospital No. 4 in Lublin, Jaczewskiego 8, 20-090 Lublin, Poland; kamil.arciszewski@usk4.lublin.pl; 8 Department of Neuroproteomics, Paracelsus Medical University, 5020 Salzburg, Austria; gert.lubec@lubeclab.com

**Keywords:** dopaminergic modulation, chronic stress, tibia, femur, growth plate

## Abstract

**Background/Objectives:** Post-traumatic stress disorder (PTSD) has been associated with negative effects on bone health, potentially leading to reduced bone mass, altered geometry, and impaired mechanical strength. However, the extent of these changes and possible pharmacological interventions remains unclear. This study aimed to assess the impact of PTSD on bone properties and evaluate the therapeutic potential of CE-123 in mitigating PTSD-induced skeletal deterioration. Additionally, we examined the effects of CE-123 on healthy bone to determine its broader influence on skeletal integrity and growth. **Methods:** We conducted an experiment using female Wistar rats divided into four groups: Control, PTSD, Control+CE-123, and PTSD+CE-123. PTSD was induced using a validated stress paradigm, and CE-123 was administered to evaluate its effects on bone properties. Morphometric, densitometric, and mechanical parameters of the tibia and femur were analyzed, along with growth plate measurements to assess potential effects on skeletal development. **Results:** PTSD led to significant reductions in bone mineral density, bone mass, and mechanical properties, particularly in cortical thickness and relative bone weight, suggesting increased bone fragility. CE-123 treatment in PTSD-exposed rats prevented some of these adverse effects but did not fully restore bone integrity. In healthy rats, CE-123 increased bone length and growth plate size, particularly in the proliferative and resting zones, indicating a stimulatory effect on bone growth. **Conclusions:** PTSD negatively affects bone structure and mechanical strength, while CE-123 shows a potential to mitigate these effects. However, its influence on healthy bones raises questions about its long-term impact on skeletal development. Further studies are needed to evaluate CE-123’s clinical applicability and safety, particularly in younger populations.

## 1. Introduction

Post-Traumatic Stress Disorder (PTSD) is a severe and debilitating mental health condition that arises following exposure to highly stressful or traumatic events, such as life-threatening situations, serious injury, or violence. It is characterized by persistent and intrusive memories of the traumatic event, avoidance of reminders, and heightened physiological arousal. PTSD not only affects mental health but also has far-reaching impacts on the body’s morpho-physiological systems [1]. The prevalence of PTSD varies across different populations, with estimates suggesting that approximately 8–10% of individuals will experience PTSD at some point in their lives [1,2]. Women are more likely than men to develop PTSD, which may be due to a combination of biological, psychological, and social factors. The severity, frequency, and duration of the traumatic event, along with individual factors such as genetic predisposition and previous psychological history, play crucial roles in the development and persistence of PTSD symptoms [3].

Research has increasingly shown that PTSD can cause neurological changes by altering brain structure and chemistry, leading to a prolonged stress response [4,5]. In addition to neurological impact, PTSD can have significant effects on musculoskeletal health. Chronic stress associated with PTSD disrupts the hypothalamic–pituitary–adrenal (HPA) axis, altering cortisol levels and potentially heightening systemic inflammation [6,7]. Elevated cortisol, a key stress hormone, can inhibit bone formation and promote bone resorption, leading to decreased bone density and compromised bone strength [8]. Moreover, chronic unpredictable mild stress (CUMS) in mice has been associated with osteoporosis due to abnormal osteoclast activity, with miR-335-3p downregulation leading to accelerated osteoclastic differentiation [9,10]. Additionally, a murine model of PTSD involving inescapable foot shock and social isolation demonstrated long-term alterations in bone physiology, including trabecular bone loss and changes in bone tissue architecture and cellular activity [11]. Furthermore, traumatic events causing PTSD have been shown to have lasting consequences on skeletal growth, with electric shocks and anxiogenic drug injections leading to reduced bone mineral content and density in young mice [12]. These physiological disruptions raise concerns about the increased risk of osteoporosis and fractures in individuals with PTSD [3].

PTSD is not exclusive to humans; it can also manifest in animals, particularly those exposed to extreme stress or traumatic events. Animals, like humans, have complex emotional and psychological responses to their environments, and traumatic experiences can lead to long-lasting behavioral and physiological changes. The conditioned fear response, where animals learn to associate a neutral stimulus with an aversive event, results in heightened defensive reactions upon subsequent exposure to the stimulus, even in the absence of the original threat. This learned fear response is a fundamental model for studying PTSD, as it mirrors the persistent and exaggerated fear responses observed in trauma-exposed individuals [13].

CE-123, a novel and highly selective dopamine transporter (DAT) inhibitor, has shown promise in enhancing cognitive functions in aged rats and improving reward-motivated tasks [14]. Additionally, studies on the dopamine transporter gene variant DAT Val559 have highlighted its role in perturbing dopamine neurotransmission and behavior, leading to compulsive behaviors and altered reward processing [15]. Furthermore, the atypical DAT inhibitor CE-123 has demonstrated the ability to counteract spatial learning deficits induced by maternal separation in rats, suggesting its potential to restore cognitive impairment and dopamine signaling, particularly in females [16]. These findings collectively support the notion that compounds like CE-123, by modulating dopaminergic signaling, could indeed hold promise in attenuating the physiological and psychological effects of conditions like PTSD, warranting further exploration in relevant models. Beyond its neurological effects, dopamine signaling plays a crucial role in bone metabolism, influencing osteoblast proliferation, differentiation, and bone remodeling. Recent studies suggest that dopamine transporter inhibition can enhance bone formation by increasing osteoblastic activity and reducing osteoclast-driven bone resorption [17]. Given that PTSD is associated with increased systemic inflammation and stress-induced bone loss, CE-123’s dopaminergic modulation may provide a dual benefit, both in mitigating PTSD-related neurophysiological effects and in preserving bone integrity by promoting bone formation and reducing resorption [18].

Our primary goal was to investigate the impact of PTSD on bone properties and to evaluate whether CE-123 could mitigate any observed skeletal deterioration. Specifically, we aimed to analyze the morphometric, densitometric, and mechanical characteristics of the tibia and femur, as well as assess changes in the growth plate structure, to determine how PTSD and CE-123 influence bone development and integrity. We hypothesized that PTSD would negatively affect bone properties by reducing bone density, strength, and structural integrity due to stress-induced hormonal imbalances, such as elevated cortisol levels, which enhance bone resorption. Additionally, we hypothesized that CE-123 treatment would counteract these detrimental effects by preserving or improving bone properties and supporting normal growth plate function. To test this, we implemented a well-structured experimental design with four groups of rats: a control group, a PTSD-induced group, and subgroups within each receiving either a placebo or CE-123. The findings from this study could contribute to a better understanding of the systemic consequences of PTSD on bone health and the potential of pharmacological interventions to prevent stress-related skeletal deterioration and growth impairments.

## 2. Materials and Methods

### 2.1. Animals

The experiment was conducted on 48 30-day-old female Wistar rats initially weighing 112–150 g (OMD, Lublin, Poland), which were randomly divided into four groups: Control, Control + CE-123, PTSD, PTSD + CE-123 (*n* = 12 per group). The animals were housed in standard laboratory conditions in cages measuring 55 cm × 33 cm × 20 cm, under a 12 h light/dark cycle, with a controlled room temperature of 21 ± 1 °C. Food (Sniff Spezialdiäten GmbH, Soest, Germany) and water were available ad libitum.

To minimize stress related to experimental handling, all rats were accustomed to human interaction by being handled for 5 min daily over five consecutive days prior to fear conditioning. All procedures were approved by the Local Ethics Committee for Experiments on Animals in Lublin (Poland) (approval no. 11/2024, approved on 26 February 2024) and were conducted in accordance with the Directive 2010/63/EU, issued on 22 September 2010. The study adhered to the ARRIVE guidelines for reporting in vivo experiments.

### 2.2. Fear Conditioning

The PTSD model was established using a Fear Conditioning system (Ugo Basile, Gemonio, Italy). During the conditioning phase, animals were placed in a soundproof chamber measuring 55 cm × 60 cm × 57 cm, equipped with a light-emitting bulb, a loudspeaker, and a floor composed of metal rods capable of delivering electric shocks. The conditioned stimulus (CS) consisted of an auditory cue emitted by the loudspeaker at an intensity of 80 dB for 28 s. Additionally, the distinct striped walls and metallic floor served as contextual cues, also acting as a conditioned stimulus.

To induce fear conditioning, the auditory cue and environmental context were paired with an aversive unconditioned stimulus (US)—a mild electric shock (1.5 mA) applied to the animals’ paws during the final 2 s of the sound signal. After a brief interval, this procedure was repeated, resulting in two CS–US pairings per training session. Following conditioning, each rat remained in the chamber for 60 s to allow memory consolidation before being returned to its home cage.

The experimental groups were divided as follows: one-half of the animals (*n* = 24) received electric shocks (stressed group), while the other half was exposed only to the conditioned stimulus without shocks (non-stressed group). All experimental procedures followed previously established protocols [19], with modifications [20].

### 2.3. Treatment

(S)-CE-123 (5-((benzhydrylsulfinyl)methyl)thiazole) (CE-123), a modafinil analog, was synthesized in the Lubec Laboratory (University of Vienna, Vienna, Austria). For drug administration, CE-123 was suspended in a vehicle consisting of 3.3% Tween 80 diluted in 0.9% NaCl. The solutions were freshly prepared ex tempore before each administration. CE-123 was administered intraperitoneally (*i.p*.) at a dose of 10 mg/kg body weight. Rats from the Control + CE-123 and PTSD + CE-123 groups received CE-123 daily, while those from the Control and PTSD groups were administered the vehicle alone (3.3% Tween 80 diluted in 0.9% NaCl), following the same procedure.

### 2.4. Growth Plate Morphology

The animals were euthanized by decapitation. Immediately after euthanasia, the left femur and left tibia were collected. Bone fragments containing the growth plate were excised from the distal femur and proximal tibia using an MBS 240/E diamond bandsaw (Proxxon GmbH, Foehren, Germany). The collected samples were initially fixed in a 4% buffered formalin solution for 24 h. Subsequently, the samples were demineralized in buffered EDTA (10%, pH 7.4). After demineralization, the specimens were dehydrated and embedded in paraffin. Each bone sample was sectioned into 3 μm thick slices using a rotary microtome (Microm HM 360, Microm, Walldorf, Germany). The sections were stained with hematoxylin and eosin (H&E) for the morphological assessment of the growth plate. Microscopic evaluation was performed using a light microscope (Olympus BX63, Olympus, Tokyo, Japan). The acquired images were analyzed using ImageJ software (v.148, National Institutes of Health, Bethesda, MD, USA). The thickness of the total growth plate and the thickness of the three zones: hypertrophic, proliferative, and resting were measured.

### 2.5. Bone Analysis

The right femur and right tibia were used for the bone analysis. Bone mineral density (BMD) and bone mineral content (BMC) were measured after the bones were thawed overnight at 4 °C using a Lunar densitometer (GE, Madison, WI, USA) and analyzed with the dual-energy X-ray absorptiometry (DXA) method. Each tibia and femur were scanned along its entire length, and BMD and BMC values were recorded for further evaluation. Following DXA measurements, the bones underwent mechanical testing using a three-point bending test to assess their structural properties. The tests were conducted on a Zwick Z010 universal testing machine (Zwick-Roell GmbH & Co., Ulm, Germany) with the span distance adjusted to match the bone length and a loading rate set at 10 mm/min. Each bone was positioned horizontally on two supports, with the mid-diaphysis serving as the loading site. During testing, load–displacement curves were generated, allowing for the determination of mechanical parameters such as yield load (F_yield_), fracture load (F_max_), stiffness, elastic work (W_yield_), and work to fracture (W_max_). These parameters were analyzed using Origin software (v. 2022, OriginLab, Northampton, MA, USA) to quantify the mechanical properties of the bones.

Once mechanical testing was completed, the bones were sectioned transversely at the mid-diaphysis using an MBS 240/E diamond bandsaw (Proxxon GmbH, Foehren, Germany). Cross-sectional cortical bone diameters were measured with a digital caliper, including transversal outer diameter (H_out_), transversal inner diameter (H_inn_), cranial–caudal outer diameter (V_out_), and cranial–caudal inner diameter (V_inn_). These measurements were used to calculate geometric parameters such as cross-sectional area (CSA), cortical index (CI), mean relative wall thickness (MRWT), and cross-sectional moment of inertia (Ix). Additionally, based on the load–deformation curves and cross-sectional measurements, the material properties of the bones were determined using standard beam-theory equations, including yield strain (Ɛ_yield_), breaking strain (Ɛ_max_), Young’s modulus, yield stress (σ_yield_), and breaking stress (σ_max_). The methods used in this study followed previously established protocols and validated biomechanical analysis techniques [21].

### 2.6. Statistical Analysis

All statistical analyses were performed using GraphPad Prism version 10.4.0 for Windows (GraphPad Software, San Diego, CA, USA). Data distribution was tested with the Shapiro–Wilk test, and Levene’s test was used to assess the homogeneity of variances. A two-way ANOVA was conducted to analyze the effects of PTSD and the CE-123 treatment and their interaction on bone-related parameters, including growth plate measurements. The statistical model included PTSD exposure (presence or absence) and CE-123 treatment (treated or untreated) as independent variables. If the assumption of homogeneity of variances was violated, Welch’s ANOVA was applied instead of the standard two-way ANOVA. Post hoc comparisons for multiple group differences were conducted using Tukey’s test following two-way ANOVA or Games–Howell’s test if Welch’s ANOVA was used. Data are reported as mean ± standard deviation (SD), with statistical significance set at *p* < 0.05.

## 3. Results

### 3.1. Animal and Bone Characteristics

**Figure 1:**
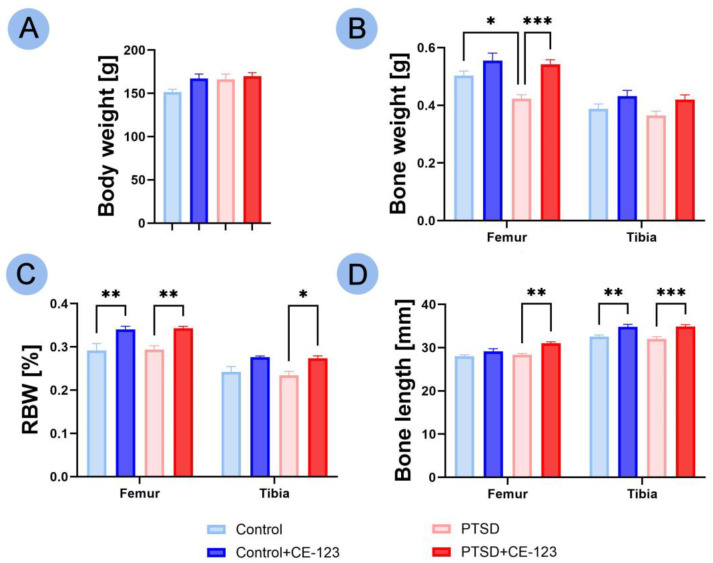
Comparison of femur and tibia morphological characteristics (body weight used as reference for other measurements) among four experimental groups: Control, PTSD, Control treated with CE-123, and PTSD treated with CE-123. Measured parameters include (**A**) body weight, (**B**) bone weight, (**C**) relative bone weight (RBW), (**D**) bone length. Asterisks (*) indicate significant differences between groups (* *p* < 0.05, ** *p* < 0.01, *** *p* < 0.001).

No significant changes in body weight were observed across all experimental groups (Figure 1A). In terms of bone weight, a significant decrease was noted in the femur of the PTSD group compared to the control group (Figure 1B; *p* < 0.05), while the PTSD group treated with CE-123 exhibited a significant increase in femur bone weight compared to the untreated PTSD group (Figure 1B; *p* < 0.001), with no significant changes observed between the control group and the control group treated with CE-123. No significant differences in bone weight were observed in the tibia among any of the groups. For the relative bone weight (RBW) in the femur, an increase was noted in the control group treated with CE-123 compared to the untreated control group (Figure 1C; *p* < 0.01), and a similar increase was observed in the PTSD group treated with CE-123 compared to the untreated PTSD group (Figure 1C; *p* < 0.01). In the tibia, an increase in RBW was observed only between the PTSD group and the PTSD group treated with CE-123, with no significant changes noted in the control groups (Figure 1C; *p* < 0.05). Regarding bone length, the femur showed a significant increase in the PTSD group treated with CE-123 compared to the untreated PTSD group (Figure 1D; *p* < 0.01). In the tibia, bone length showed a significant increase in the control group treated with CE-123 compared to the untreated control group, as well as between the PTSD group treated with CE-123 and the untreated PTSD group (Figure 1D; *p* < 0.01 and *p* < 0.001, respectively).

### 3.2. Bone Properties

**Figure 2:**
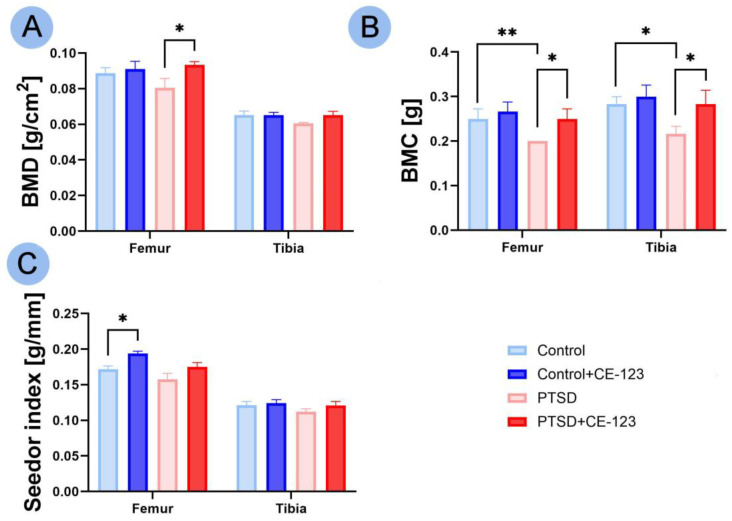
Comparison of femur and tibia bone properties among four experimental groups: Control, PTSD, Control treated with CE-123, and PTSD treated with CE-123. Measured parameters include (**A**) bone mineral density (BMD), (**B**) bone mineral content (BMC), (**C**) Seedor index. Asterisks (*) indicate significant differences between groups (* *p* < 0.05, ** *p* < 0.01).

In BMD, the only significant difference observed was an increase in the femur of the PTSD group treated with CE-123 compared to the untreated PTSD group (Figure 2A; *p* < 0.05). For BMC, a decrease was observed in the PTSD group compared to the control group, while an increase was noted in the PTSD group treated with CE-123 compared to the untreated PTSD group for both the femur (Figure 2B; *p* < 0.01 and *p* < 0.05, respectively) and tibia (Figure 2B; *p* < 0.05). In the Seedor index, a significant increase was observed only in the femur between the control group and the control group treated with CE-123 (Figure 2C; *p* < 0.05).

### 3.3. Geometrical Properties

**Figure 3:**
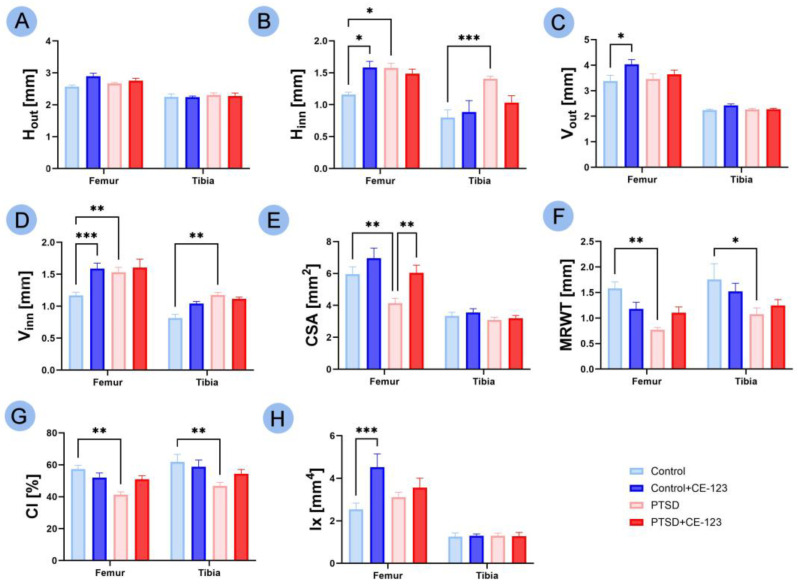
Comparison of femur and tibia geometrical properties among four experimental groups: Control, PTSD, Control treated with CE-123, and PTSD treated with CE-123. Measured parameters include (**A**) transversal outer diameter (H_out_), (**B**) transversal inner diameter (H_inn_), (**C**) cranial–caudal outer diameter (V_out_), (**D**) cranial–caudal inner diameter (V_inn_), (**E**) mid-diaphysis cross-sectional area (CSA), (**F**) mean relative wall thickness (MRWT), (**G**) cortical index (CI), (**H**) cross-sectional moment of inertia (Ix). Asterisks (*) indicate significant differences between groups (* *p* < 0.05, ** *p* < 0.01, *** *p* < 0.001).

No significant changes were observed in the H_out_ across all groups. In the H_inn_, an increase was observed in the femur of both the PTSD group (Figure 3B; *p* < 0.05) and the control group treated with CE-123 (Figure 3B; *p* < 0.05) compared to the control group, while in the tibia, a significant increase was noted between the control group and the PTSD group (Figure 3B; *p* < 0.05). For V_out_, an increase was observed only in the femur of the control group treated with CE-123 compared to the untreated control group (Figure 3C; *p* < 0.05). In V_inn_, an increase was observed in the femur of both the PTSD group (Figure 3D; *p* < 0.001) and the control group treated with CE-123 (Figure 3D; *p* < 0.001) compared to the control group, while in the tibia, a significant increase was noted between the control group and the PTSD group (Figure 3D; *p* < 0.01). In CSA of the femur, there was a decrease in the PTSD group compared to the control group (Figure 3E; *p* < 0.01), followed by an increase in the PTSD group treated with CE-123 compared to the untreated PTSD group (Figure 3E; *p* < 0.01). The MRWT showed a decrease in both the femur and tibia between the control and PTSD groups (Figure 3F; *p* < 0.01 and *p* < 0.05, respectively). Similarly, the CI also showed a decrease in both the femur and tibia between the control and PTSD groups (Figure 3G; *p* < 0.01 for both). In the Ix of the femur, a significant increase was observed between the control group and the control group treated with CE-123 (Figure 3H; *p* < 0.001).

### 3.4. Mechanical Properties

**Figure 4:**
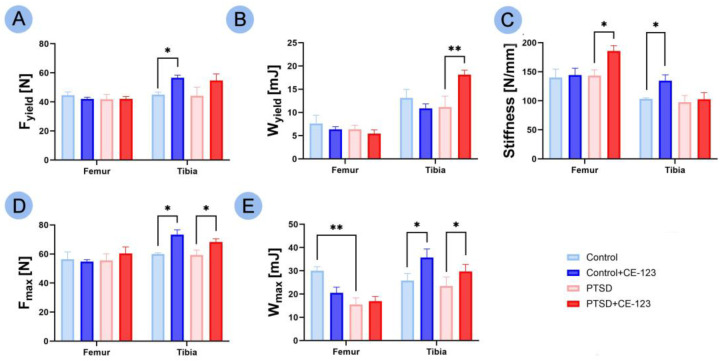
Comparison of femur and tibia mechanical properties among four experimental groups: Control, PTSD, Control treated with CE-123, and PTSD treated with CE-123. Measured parameters include (**A**) yield force (F_yield_), (**B**) elastic work (W_yield_), (**C**) stiffness, (**D**) breaking force (F_max_), (**E**) breaking work (W_max)_. Asterisks (*) indicate significant differences between groups (* *p* < 0.05, ** *p* < 0.01).

An increase in tibia F_yield_ was observed between the control group and the control group treated with CE-123 (Figure 4A; *p* < 0.05). A substantial increase in W_yield_ in the tibia was noted between the PTSD group and the PTSD group treated with CE-123 (Figure 4B; *p* < 0.01). In terms of stiffness, an increase was observed in the femur between the PTSD group and the PTSD group treated with CE-123 (Figure 4C; *p* < 0.05), and in the tibia between the control group and the control group treated with CE-123 (Figure 4C; *p* < 0.05). F_max_ in the tibia showed an increase between both the control and PTSD groups and their respective CE-123 treated groups (Figure 4D; *p* < 0.05 for both). A decrease in W_max_ in the femur was observed between the control and PTSD groups (Figure 4E; *p* < 0.01), while an increase in W_max_ in the tibia was noted between both the control and PTSD groups and their respective CE-123-treated groups (Figure 4E; *p* < 0.05 for both).

### 3.5. Bone Material Properties

**Figure 5:**
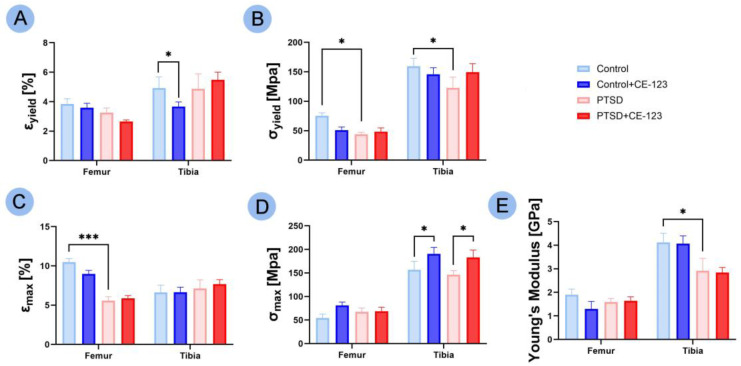
Comparison of femur and tibia bone material properties among four experimental groups: Control, PTSD, Control treated with CE-123, and PTSD treated with CE-123. Measured parameters include (**A**) yield strain (Ɛ*_yield_*), (**B**) yield stress (σ_yield_), (**C**) breaking strain (Ɛ_max_), (**D**) breaking stress (σ_max_), (**E**) Young’s modulus. Asterisks (*) indicate significant differences between groups (* *p* < 0.05, *** *p* < 0.001).

A decrease in Ɛ_yield_ was observed in the tibia between the control group and the control group treated with CE-123 (Figure 5A; *p* < 0.05). Both the femur and tibia showed a decrease in σ_yield_ between the control and PTSD groups (Figure 5B; *p* < 0.05). A significant decrease in Ɛ_max_ in the femur was noted between the control and PTSD groups (Figure 5C; *p* < 0.001). An increase in σ_max_ in the tibia was observed between both the control and PTSD groups and their respective CE-123 treated groups (Figure 5D; *p* < 0.05 for both). Finally, a decrease in Young’s modulus in the tibia was seen between the control and PTSD groups (Figure 5E; *p* < 0.05).

### 3.6. Growth Plate

**Figure 6:**
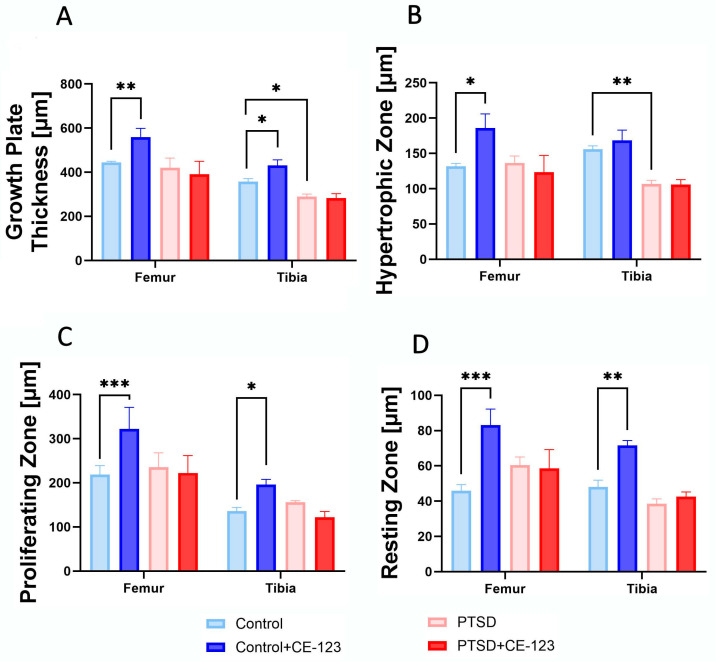
A comparison of femur and tibia bone growth plate morphology among the four experimental groups: Control, PTSD, Control treated with CE-123, and PTSD treated with CE-123. The measurements of the growth plate include (**A**) overall growth plate thickness, (**B**) hypertrophic zone thickness, (**C**) proliferating zone thickness, and (**D**) resting zone thickness. Asterisks (*) indicate significant differences between the groups (* *p* < 0.05, ** *p* < 0.01, *** *p* < 0.001).

**Figure 7:**
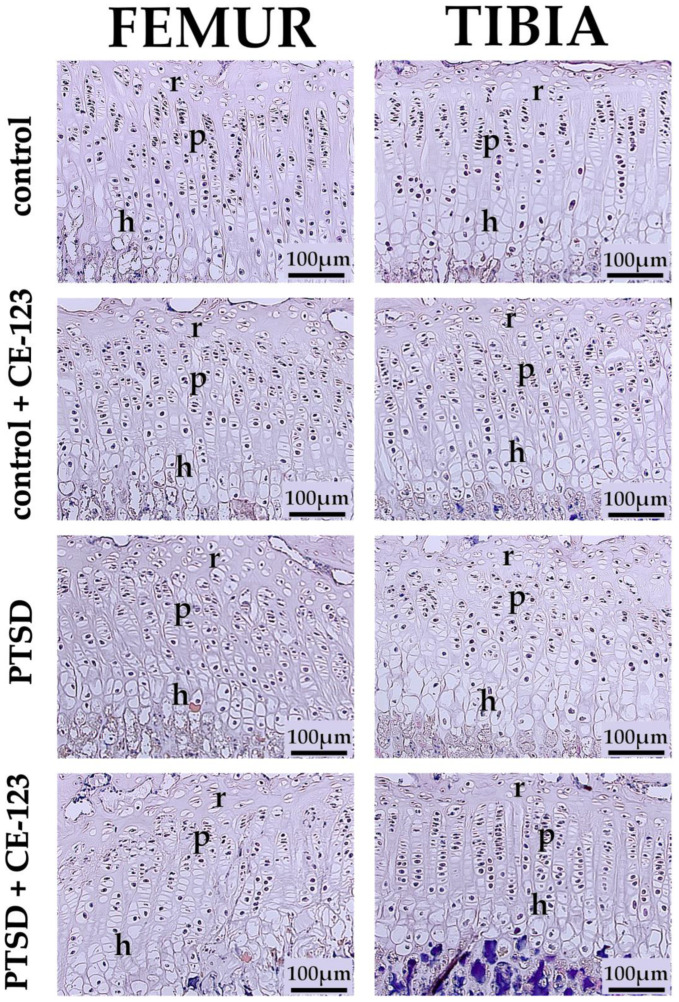
Representative microphotographs of the femoral growth plate (first column) and tibial growth plate (second column) in the Control group (first row), Control + CE-123 group (second row), PTSD group (third row), and PTSD + CE-123 group (fourth row). Letters indicate specific growth plate zones: r—resting zone; p—proliferative zone; h—hypertrophic zone. Scale bar = 100 µm.

Growth plate thickness was significantly increased in the femur of the CE-123-treated control group compared to the untreated control group (*p* < 0.001), while in the tibia, a similar increase was observed (*p* < 0.05). Additionally, PTSD-exposed rats showed a significant reduction in tibial growth plate thickness compared to the control group (Figure 6A; *p* < 0.05). In the hypertrophic zone, a significant increase was observed in the femur of the CE-123-treated control group compared to the untreated control group (*p* < 0.05). Conversely, in the tibia, the PTSD group exhibited a significant decrease compared to the control (Figure 6B; *p* < 0.01). For the proliferating zone, CE-123 treatment resulted in a significant increase compared to the control group in both the femur and tibia (Figure 6C; *p* < 0.001 and *p* < 0.05, respectively). Similarly, in the resting zone, CE-123 treatment led to a significant increase compared to the control group in both the femur and tibia (Figure 6D; Figure 7; *p* < 0.001 and *p* < 0.01).

## 4. Discussion

The present study investigated the effects of PTSD on bone biomechanics and morphology, as well as the potential of CE-123 to mitigate PTSD-induced skeletal changes. Our findings demonstrate that PTSD significantly affects femoral and tibial properties, leading to reduced bone mass, altered geometry, and compromised mechanical integrity. Treatment with CE-123 ameliorated some of these deleterious effects, suggesting a potential role for this compound in preserving bone health in PTSD-affected individuals. These findings are consistent with previous research indicating that chronic stress and PTSD contribute to osteoporosis and impaired bone regeneration [22,23].

PTSD is recognized as a significant factor influencing skeletal health through multiple pathways. One primary mechanism involves prolonged dysregulation of the autonomic nervous system and the HPA axis, leading to the altered secretion of glucocorticoids and catecholamines [24,25]. These hormonal changes contribute to increased osteoclastic activity and suppressed osteoblast function, which could explain the reduced bone weight and cortical thickness observed in our study [26,27]. Additionally, PTSD-induced alterations in bone remodeling likely contributed to the increased inner diameter and reduced MRWT both in the femur and tibia of PTSD-exposed rats, as chronic stress has been associated with compromised bone structure and increased porosity [28]. The overall reduction in bone mass seen in the PTSD group aligns with previous findings linking stress-induced inflammation and HPA axis dysfunction to osteoporosis-like changes in animal models [29]. Additionally, PTSD can influence bone structure through systemic inflammatory pathways. Chronic stress is associated with increased levels of pro-inflammatory cytokines, such as tumor necrosis factor-alpha and interleukin-6, which are known to stimulate osteoclast differentiation and suppress osteoblast proliferation [30,31]. These cytokine-mediated alterations contribute to trabecular bone loss and increased cortical porosity, making bones more susceptible to fractures. The observed reductions in breaking strain and Young’s modulus in PTSD-exposed rats may reflect these changes, as chronic inflammation has been shown to weaken bone material properties by disrupting collagen organization and mineralization [32]. Stress-induced bone fragility has been demonstrated in experimental PTSD models, where prolonged exposure to severe stressors resulted in significant reductions in bone strength, stiffness, and yield strain [33]. The reduction in yield strain observed in our study further supports this connection, indicating that chronic stress not only reduces bone mass but also impairs its ability to withstand mechanical loads. These findings reinforce the role of systemic inflammation as a key factor in PTSD-induced skeletal deterioration and highlight the importance of interventions targeting inflammatory pathways to mitigate these effects.

CE-123’s ability to partially reverse PTSD-induced skeletal deterioration may be linked to its role in modulating dopaminergic pathways. Dopamine plays a crucial role in bone metabolism, influencing osteoblast proliferation and differentiation through D1 and D2 receptor-mediated signaling [17,34]. Our findings indicate that CE-123 treatment significantly increased bone mass and mechanical strength in PTSD-exposed rats, suggesting a protective effect against stress-induced bone loss. Studies have shown that DAT inhibition can enhance neurogenesis and reduce inflammatory cytokine production [35]. Given that PTSD is associated with increased pro-inflammatory markers, CE-123’s role in reducing systemic inflammation could indirectly benefit bone homeostasis by mitigating excessive osteoclastic activity [36]. Conversely, CE-123 has been shown to influence bone metabolism and structural integrity even in the absence of PTSD-related stressors. The observed increase in RBW, the Seedor index, inner diameter, and Ix suggests a more complex direct interaction between CE-123 and osteogenic pathways. These effects may stem from CE-123’s role in modulating dopaminergic signaling, which has been implicated in osteoblast proliferation and bone matrix deposition [17]. The increase in RBW and the Seedor index suggests an enhancement of cortical bone mass and density, potentially improving bone strength and resistance to mechanical loading [9]. The enlargement of the inner diameter and increased Ix indicate the potential remodeling of the bone architecture, which could be beneficial in mature individuals by optimizing load distribution and resistance to torsional forces [27]. However, in young, growing animals, these changes could disrupt normal bone development by altering the balance between bone resorption and formation during growth. A wider inner diameter may lead to a thinner cortical bone, potentially reducing overall bone strength and increasing susceptibility to fractures under mechanical stress. The increased bone mass and altered geometric parameters may lead to premature skeletal maturation, potentially disrupting normal growth plate activity and longitudinal bone development [37]. Early closure of the growth plates due to enhanced osteoblastic activity and increased mineral deposition could result in stunted growth or abnormal bone morphology [38]. Additionally, excessive cortical thickening may compromise bone flexibility, increasing the risk of stress fractures in high-impact scenarios [37].

Furthermore, PTSD-related disruptions in the endocrine system, particularly in the growth hormone (GH) and insulin-like growth factor-1 (IGF-1) axis, can also impair bone formation and growth. Chronic stress has been linked to reduced GH secretion, which in turn leads to compromised chondrocyte function and delayed endochondral ossification [38]. This effect is particularly concerning in younger populations, where PTSD-induced stress can result in stunted growth and long-term deficits in skeletal integrity [37]. Studies have confirmed that individuals exposed to early-life trauma exhibit lower bone mineralization and increased fracture risk later in life [38]. One of the key findings of our study was the significant impact of PTSD on growth plate integrity, which in later life, could lead to a reduction in femoral and tibial length. PTSD significantly reduced overall growth plate thickness, particularly through a decrease in the hypertrophic zone, which is critical for bone elongation. This suggests that PTSD impairs normal endochondral ossification, likely due to increased chondrocyte apoptosis and inflammatory signaling [39]. Interestingly, CE-123 did not restore growth plate thickness in PTSD-exposed rats but significantly increased it in healthy rats, primarily through an expansion of the proliferative and resting zones. This indicates that CE-123 promotes chondrocyte proliferation and delays differentiation, possibly by modulating dopaminergic signaling pathways that influence bone growth [25,40]. The increase in the proliferative and resting zones in CE-123-treated rats suggests enhanced early-stage chondrocyte activity, which could lead to longer bones if sustained. In our study, we observed significantly longer femoral and tibial bones in rats treated with CE-123, supporting the idea that this compound promotes longitudinal bone growth. However, the lack of change in the hypertrophic zone raises concerns about whether the chondrocytes fully mature, a necessary step for proper bone mineralization [41]. The reduction in the proliferative zone in PTSD rats treated with CE-123 compared to PTSD-alone rats suggests that CE-123 may have different effects under stress conditions, potentially altering the normal adaptive response of the growth plate. While increased growth plate thickness in healthy animals might be beneficial for growth, prolonged alterations in chondrocyte activity could lead to abnormal bone development or premature growth plate closure [42]. These findings indicate that while CE-123 has potential benefits for bone growth, its long-term effects on skeletal maturation and integrity require further investigation, especially in younger populations.

Bones exhibit site-specific differences in their structural, mechanical, and material properties, which influence their response to physiological stress and pharmacological interventions. In our study, BMC remained similar between the femur and tibia, but the mechanical and material properties were more significantly altered in the tibia. These findings align with research showing that the tibia, which has a higher proportion of trabecular bone, is more metabolically active and thus more responsive to systemic influences, including stress and pharmacological treatment [43,44]. Conversely, changes in geometric properties were more pronounced in the femur, likely due to its higher cortical bone content and primary adaptation to mechanical loading [45]. These distinctions suggest that while the tibia may be more vulnerable to systemic metabolic disruptions due to its high bone turnover rate, the femur undergoes structural modifications that are more reflective of long-term mechanical adaptation. This divergence in response patterns highlights the complexity of skeletal adaptation, where different regions of the skeleton prioritize either metabolic homeostasis or biomechanical resilience depending on their functional demands. Understanding these site-specific differences is critical when evaluating the effects of stress and pharmacological interventions on bone health, as treatments that benefit one skeletal site may not exert the same effects on another.

## 5. Conclusions

In conclusion, this study confirms the detrimental effects of PTSD on bone health and highlights CE-123 as a potential protective agent. CE-123 improved bone mass and strength in PTSD-affected individuals, suggesting a therapeutic role in counteracting stress-induced skeletal deterioration. Additionally, its effects on healthy bones indicate a broader influence on bone metabolism, which may have implications for growth and adaptation. These findings support further investigation into CE-123 as a potential treatment for PTSD-related osteoporosis and bone fragility, with future studies needed to assess its long-term safety and clinical applicability.

## Data Availability

All data are available within the manuscript.

## Abbreviations

The following abbreviations are used in this manuscript:

PTSD	Post-traumatic stress disorder
HPA	Hypothalamic–pituitary–adrenal
CUMS	Chronic unpredictable mild stress
DAT	Dopamine transporter
CS	The conditioned stimulus
BMD	Bone mineral density
BMC	Bone mineral content
F_yield_	Yield force
F_max_	Breaking force
W_yield_	Elastic work
W_max_	Breaking work
H_out_	Transversal outer diameter
H_inn_	Transversal inner diameter
V_out_	Cranial–caudal outer diameter
V_inn_	Cranial–caudal inner diameter
CSA	Cross-sectional area
MRWT	Mean relative wall thickness
CI	Cortical index
Ix	Moment of inertia
Ɛ_yield_	Yield strain
Ɛ_max_	Breaking strain
σ_yield_	Yield stress
σ_max_	Breaking stress
RBW	Relative Bone Weight
GH	Growth hormone
